# Impairment of Glucose Metabolism and Suppression of Stemness in MCF-7/SC Human Breast Cancer Stem Cells by Nootkatone

**DOI:** 10.3390/pharmaceutics14050906

**Published:** 2022-04-21

**Authors:** Yen Thi-Kim Nguyen, Ngoc Bao To, Vi Nguyen-Phuong Truong, Hee Young Kim, Meran Keshawa Ediriweera, Yoongho Lim, Somi Kim Cho

**Affiliations:** 1 Interdisciplinary Graduate Program in Advanced Convergence Technology and Science, Jeju National University, Jeju 63243, Korea; ntkyen.hcmus@gmail.com (Y.T.-K.N.); tobaongoc.hcmus@gmail.com (N.B.T.); phuongvi.truongnguyen@gmail.com (V.N.-P.T.); hi.khyoung@gmail.com (H.Y.K.); 2 Subtropical—Tropical Organism Gene Bank, Jeju National University, Jeju 63243, Korea; mk.ediriweera@gmail.com; 3 Department of Biochemistry and Molecular Biology, Faculty of Medicine, University of Colombo, Colombo 00300, Sri Lanka; 4 Department of Biological Sciences, Konkuk University, Seoul 05029, Korea; yoongho@konkuk.ac.kr

**Keywords:** breast cancer stem cells, glycolysis, oxidative phosphorylation, cancer metabolism, nootkatone, AMPK

## Abstract

Targeting cancer stem cell metabolism has emerged as a promising therapeutic strategy for cancer treatment. Breast cancer stem cells (BCSCs) exert distinct metabolism machinery, which plays a major role in radiation and multidrug resistance. Therefore, exploring the mechanisms involved in energy utilization of BCSCs could improve the effectiveness of therapeutic strategies aimed at their elimination. This study was conducted to clarify the glucose metabolism machinery and the function of nootkatone, a bioactive component of grapefruit, in regulating glucose metabolism and stemness characteristics in human breast carcinoma MCF-7 stem cells (MCF-7SCs). In vivo experiments, transcriptomic analysis, seahorse XF analysis, MTT assay, Western blotting, mammosphere formation, wound healing, invasion assay, flow cytometric analysis, reverse transcription-quantitative polymerase chain reaction, and in silico docking experiments were performed. MCF-7SCs showed a greater tumorigenic capacity and distinct gene profile with enrichment of the genes involved in stemness and glycolysis signaling pathways compared to parental MCF-7 cells, indicating that MCF-7SCs use glycolysis rather than oxidative phosphorylation (OXPHOS) for their energy supply. Nootkatone impaired glucose metabolism through AMPK activation and reduced the stemness characteristics of MCF-7SCs. In silico docking analysis demonstrated that nootkatone efficiently bound to the active site of AMPK. Therefore, this study indicates that regulation of glucose metabolism through AMPK activation could be an attractive target for BCSCs.

## 1. Introduction

Breast cancer is the most common cancer among women worldwide, accounting for nearly 11.7% of new cancer cases in 2020; its incidence is projected to increase by more than 46% by 2040 [1]. Many treatment strategies have been established for breast cancer, including surgery, chemotherapy, radiotherapy, and hormone therapy. However, these conventional treatments have limitations due to resistance to chemotherapy and radiotherapy, recurrence, metastasis, and weak immunological surveillance. The main cause of breast cancer treatment failure can be attributed to cancer stem cells (CSCs) [2]. Therefore, eliminating the CSC population is considered a potential approach to improve the effectiveness of current therapeutic strategies. In this study, we utilized MCF-7SCs as a model for examining the characteristics of breast cancer stem cells and looking for novel therapeutics that can target breast cancer stem cells. In a previous study, we revealed that MCF-7SCs were the small population (4.39%) that were sorted from MCF-7 cells using the CD44^+^ and CD24^−^ marker. In comparison to its parental cells MCF-7, MCF-7SCs exert enrichment of stemness characteristics, as evidenced by the enhancement of the CD44^+^/CD24^−^ population, higher ALDH level, increased mammosphere formation capacity, reduced ROS levels, overexpressed drug efflux proteins (MDR1 and MRP1), and enhanced migration and invasion capabilities [3,4].

One hallmark of cancer cells is their ability to proliferate rapidly. Cellular metabolic processes are adept to support high rates of proliferation [5]. Cancer cells are largely dependent on “aerobic glycolysis” rather than oxidative phosphorylation (OXPHOS) for energy production, regardless of oxygen availability; this is known as the Warburg effect [6]. However, the ability of different types of CSCs to meet their energy needs via glycolysis or OXPHOS varies [7]. Although stem cells primarily use glycolysis rather than OXPHOS for energy production [8,9], in pancreatic and ovarian CSCs, energy is produced by mitochondrial oxidative metabolism [7]. As such, the overall data on CSC metabolism are inconsistent and contradictory. Further exploration of the mechanisms of energy use of breast cancer stem cells (BCSCs) is needed for the development of new breast cancer therapeutics [10,11].

One of the most well-known glucose metabolism regulators is the AMP-activated protein kinase (AMPK), which is a highly conserved serine/threonine protein kinase that consists of a catalytic α-subunit, scaffolding β-subunit, and regulatory γ-subunit [12]. Phosphorylated AMPK (p-AMPK) induces mTOR inhibition by activating the TSC1/2 complex, which regulates cell metabolism, survival, and development [13]. AMPK activation interferes with the growth of cancer cells and tumors by inhibiting MAPK, PI3K-AKT, and mTOR signaling [14,15,16]. However, the role of AMPK in metabolic control of BCSCs has yet to be determined. Nootkatone is a natural chemical produced in grapefruit that reduces diet-induced obesity and stimulates energy metabolism by activating AMPK [17,18]. Nootkatone may also act as an anticancer agent in lung and colorectal tumors [19,20]. In this study, we investigated the hypothesis that regulation of glucose metabolism by AMPK activation could be the mechanism underlying the effect of nootkatone on BCSCs. We report for the first time that nootkatone regulates glucose metabolism and suppresses the stemness characteristics of BCSCs.

## 2. Materials and Methods

### 2.1. Cell Culture

Human MCF-7 cells were purchased from the American Type Culture Collection (Rockville, MD). The BCSC cell line MCF-7SC was sorted from MCF-7 cells using the CD44^+^ and CD24^−^ markers and characterized as described previously [3,4]. MCF-7 cells and MCF-7SCs were cultured in Dulbecco’s modified Eagle’s medium (DMEM) and Roswell Park Memorial Institute (RPMI) medium, respectively, supplemented with 10% heat-activated fetal bovine serum (FBS), 100 U/mL penicillin, and 100 µg/mL streptomycin. All cells were maintained at 37 °C in a 5% CO_2_ atmosphere. Cells were sub-cultured upon reaching 70–80% confluence.

### 2.2. Tumor Xenograft Experiments

Six-week-old athymic BALB/c male nude mice (*n* = 4/group) were maintained under conditions recommended by the Institutional Animal Care and Use Committee of Jeju National University (IACUC, Jeju, Korea), which approved the animal experiments (approval no. 2019-0006). All mice were housed under pathogen-free conditions and a 12/12 h light/dark cycle and had free access to food and water at 20–25 °C. MCF-7 and MCF-7SCs (5 × 10^6^ per tumor) were injected into the fat pad. Cells were resuspended in 50 µL of Matrigel (Sigma-Aldrich, St. Louis, MO, USA) mixed with 50 µL of phosphate-buffered saline (PBS). Tumor volume and mouse weight were measured on days 10, 17, 24, 27, 29, and 31 after injection. After 31 days, mice were euthanized, and the tumors were collected and weighed.

### 2.3. Transcriptomic Analysis

Transcriptomic analysis was performed as described previously [21]. Total RNA from MCF-7 and MCF-7SCs was isolated using a TRIzol kit (Invitrogen, Carlsbad, CA, USA) and subjected to RNA sequencing (RNA-seq) by Macrogen (Seoul, Korea). Signaling pathways were investigated using the Kyoto Encyclopedia of Genes and Genomes (KEGG) Automatic Annotation Server (KAAS).

### 2.4. Cell Viability Assay

Cells (2 × 10^4^/mL) were seeded in 96-well dishes and treated with nootkatone (Sigma-Aldrich) (12.5–200 µM). Following 24 and 48 h of incubation, 100 µL of MTT (0.5 mg/mL) was added to each well, with further incubation for 2–3 h at 37 °C. Then, 150 µL of dimethyl sulfoxide (DMSO) was added to dissolve the formazan crystals. The absorbance at 570 nm was determined using a microplate reader (Tecan Group, Ltd., Salzburg, Austria).

### 2.5. Cell Invasion Assay

A Transwell system with 24-well plates (Corning, Corning, NY, USA) was used to assess the invasive ability of cells. The upper chambers were coated with 1 mg/mL Matrigel and incubated at 37 °C for 20 min. Following incubation, MCF-7SCs (1.5 × 10^5^/well) were seeded in serum-free RPMI in the coated upper chambers and supplemented with nootkatone (0, 50 μM). The lower chamber was filled with RPMI supplemented with 10% FBS. After incubation for 24 h at 37 °C, cells were fixed with 4% paraformaldehyde followed by methanol at room temperature for 20 min. Finally, the cells were stained with 2% crystal violet for 30 min at room temperature and observed under a phase-contrast microscope (magnification, 100×).

### 2.6. Wound Healing Assay

MCF-7SCs (1 × 10^5^/well) were seeded into 6-well cell culture plates and incubated for 24 h. Upon reaching 90% confluence, a 200 µL sterile pipette tip was used to make scratches. Next, cells were rinsed with PBS to remove detached cells, followed by exposure (or not) to nootkatone (0, 12.5, 25, 50 μM) in RPMI containing 5% FBS [22]. After incubation for 24 h, the wound areas were photographed using an inverted phase-contrast microscope (magnification, 40×).

### 2.7. Flow Cytometric Analysis of the CD44^+^/CD24^−^ Population

Cells (1 × 10^6^/mL) were seeded into 60 mm plates and treated (or not) with nootkatone. After 24 h of incubation, cells were suspended in 100 μL of immunofluorescence staining buffer supplemented with a PE-conjugated anti-human CD24 antibody (cat. no. 555428; BD Pharmingen, San Diego, CA, USA) and FITC-conjugated anti-human CD44 antibody (cat. no. 555478; BD Pharmingen) and incubated for 10 min at 4 °C. Next, cells were rinsed with PBS. A FACSCalibur flow cytometer (Becton Dickinson, Franklin Lakes, NJ, USA) and BD FACSDiva™ Software (BD Biosciences, San Jose, CA, USA) were used to analyze the CD44^+^/CD24^−^ cell population at the Bio-Health Materials Core Facility of Jeju National University.

### 2.8. ALDEFLUOR Assay

Aldehyde dehydrogenase (ALDH) activity was evaluated using an Aldefluor assay kit (cat. no. #01700; StemCell Technologies, Vancouver, BC, Canada), as described previously [3]. Cells (3 × 10^4^/mL) were loaded into 60 mm dishes. Then, the cells were treated with nootkatone (50 μM) for 24 h and subjected to Aldefluor assay using BD FACSDiva™ Software (BD Biosciences). The negative control was diethylaminobenzaldehyde (DEAB), a specific inhibitor of ALDH. ALDH-positive cells were analyzed on a FACSCalibur flow cytometer at the Bio-Health Materials Core Facility.

### 2.9. Mammosphere Formation Assay

Cells (2 × 10^4^/mL) were placed individually in ultralow-attachment dishes filled with complete MammoCult Human Medium (cat. no. #05620; StemCell Technologies) and exposed (or not) to nootkatone (0, 50, 100, or 200 μM). On day 7, mammospheres > 60 μm in diameter were observed under a phase-contrast microscope (magnification, 100×).

### 2.10. Reverse Transcription-Quantitative Polymerase Chain Reaction

Total RNA was extracted from cancer cells using TRIzol (Invitrogen) reagent and reversed-transcribed into cDNA using ImProm-II^TM^ Reverse Transcriptase (cat. no. A3802; Promega, Madison, WI, USA) following the manufacturer’s instructions. Then, two-step quantitative real-time PCR was performed (Thermal Cycler Dice Real-Time System; Takara, Shiga, Japan) using a TOPreal™ qPCR 2*×* PreMIX kit (Enzynomics, Daejeon, Korea). The cycling conditions were as follows: initial hold at 95 °C for 15 min, and 40 cycles at 95 °C for 10 s and 60 °C for 30 s. A dissociation curve was generated (95 °C for 15 s, 60 °C for 30 s, and 95 °C for 15 s). The real-time PCR primers are listed in Table S1. GAPDH was used as an endogenous control. Expression levels were calculated using the 2^−ΔΔCq^ method. Data were represented after three-time biological and three-time technical replicates [23]. 

### 2.11. Western Blot Analysis

Cell lysates were prepared using radioimmunoprecipitation assay (RIPA) buffer Thermo Fisher Scientific, Waltham, MA, USA) and quantified by BCA assay. Proteins (20–40 µg) were resolved by sodium dodecyl sulfate polyacrylamide gel electrophoresis (7.5–15% gels) and electrophoretically transferred to a PVDF membrane. After blocking with 5% skim milk at 4 °C overnight, the membranes were incubated with primary antibodies at 4 °C overnight. Primary antibodies were purchased from Cell Signaling Technology (Beverly, MA, USA), including anti-AMPK (#2532S), anti-p-AMPK (#2535S), anti-mTOR (#2983S), anti-p-mTOR (#2971S), anti-STAT3 (#9139S), anti-p-STAT3 (#9145S), and anti-GAPDH (#2118S). Except for the anti-GAPDH primary antibody (1:7000 dilution), the primary antibodies were diluted 1:1000. Horseradish peroxidase-conjugated (HRP) goat anti-rabbit (cat. no. PI-1000-1; Vector Laboratories, Burlingame, CA, USA) or anti-mouse (cat. no. PI-2000-1 Vector Laboratories) immunoglobulin G (IgG) secondary antibody was diluted 1:5000 before use. The BS ECL Plus Kit (Biosesang, Seongnam, Korea) was used to detect signals. ImageJ software (version 1.53; NIH, Bethesda, MD, USA) was used to quantify band intensities.

### 2.12. Docking

In silico docking experiments were performed on an Intel Core 2 Quad Q6600 (2.4 GHz) Linux PC with SYBYL 7.3 (Tripos, St. Louis, MO, USA) [24]. The binding site was determined using the LigPlot program [25], and 3D images were generated using the PyMOL program (PyMOL Molecular Graphics System, version 2.0; Schrödinger, LLC. Portland, OR, USA).

### 2.13. XF Seahorse Analysis

The oxygen consumption rate (OCR) and extracellular acidification rate (ECAR) were measured using an XF-24 Extracellular Flux Analyzer (Seahorse Bioscience, North Billerica, MA, USA). Cells were seeded in a XF24 plate at 10^4^/well. After 24 h, cells were treated (or not) with nootkatone (50 or 100 μM) in RPMI medium supplemented with 10% FBS. After 24 h, the original medium was replaced with warm Seahorse medium (XF Assay Base Medium, pH 7.4) supplemented with 10 mM glucose, 1 mM pyruvate, 2 mM L-glutamine, and 2 mM L-glutamine for OCR and ECAR measurements. Cells were treated with 2 μM oligomycin, 1 μM FCCP, and 1 μM rotenone/2 μM antimycin A at the indicated times for OCR measurement, and with 10 mM glucose, 1 μM oligomycin, and 50 mM 2-deoxy-D-glucose (2-DG) at the indicated times for ECAR measurement.

### 2.14. Statistical Analysis

Data are presented as the mean ± standard deviation of at least three replicates. Student’s *t*-test and Prism software (version 8.1; GraphPad Software, Inc., La Jolla, CA, USA) were used to analyze the data. A *p*-value < 0.05 was considered indicative of statistical significance.

## 3. Results

### 3.1. MCF-7SCs Had Greater Tumorigenicity Than MCF-7 Cells

**Figure 1:**
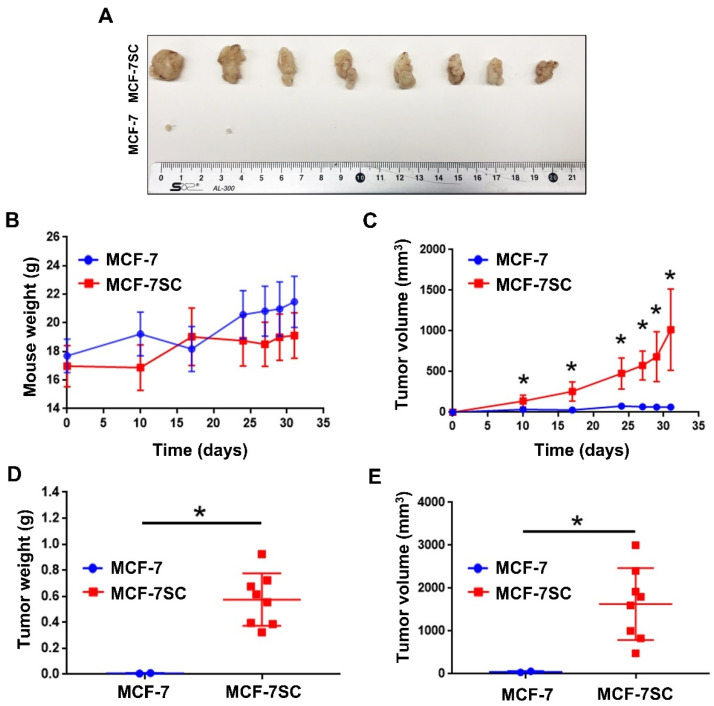
MCF-7SCs exerted higher tumorigenic capacity. (**A**) Representative tumors isolated from BALB/C nude mice injected with MCF-7 cells and MCF-7SCs (5 × 10^6^ cells per tumor). (**B**) Weight of BALB/C nude mice after injection of MCF-7 cells and MCF-7SCs at days 10, 17, 24, 27, 29, and 31. (**C**) Tumor volume at days 10, 17, 24, 27, 29, and 31. (**D**) Tumor weight and (**E**) tumor volume at day 31 after euthanizing the mice; * *p* < 0.05 vs. control; statistical values represent data from three biologically independent experiments.

MCF-7SCs possess stemness properties [3]. To confirm this in vivo, MCF-7 and MCF-7SCs were injected into immunodeficient BALB/C nude mice to compare in vivo tumorigenic ability. As shown in Figure 1A, two tumors were found in mice injected with MCF-7 cells, whereas tumors were formed at all injection sites in mice injected with MCF-7SCs. The tumors formed by MCF-7SCs were significantly larger than those formed by MCF-7 cells. Moreover, MCF-7SCs metastasized to the liver and spleen of mice, indicating higher potent tumorigenic capacity in vivo (Figure S1). The changes in body weight observed post-injection were maintained throughout the study (Figure 1B). As shown in Figure 1C, MCF-7SCs could form the tumors within 10 days after injection, and all tumors remained for up to 31 days. However, parental MCF-7 cells formed four tumors within 10 days post-injection, which decreased to two tumors by 31 days post-injection (Figure 1C). Notably, at the end of the experiment, the weight and volume of tumors derived from mice injected with MCF-7SCs were significantly higher than those derived from MCF-7 cells (Figure 1D,E). Overall, these results indicated that MCF-7SCs had greater tumorigenic potential compared to parental MCF-7 cells.

### 3.2. Transcriptomic Analysis of MCF-7SCs and MCF-7 Cells

**Figure 2:**
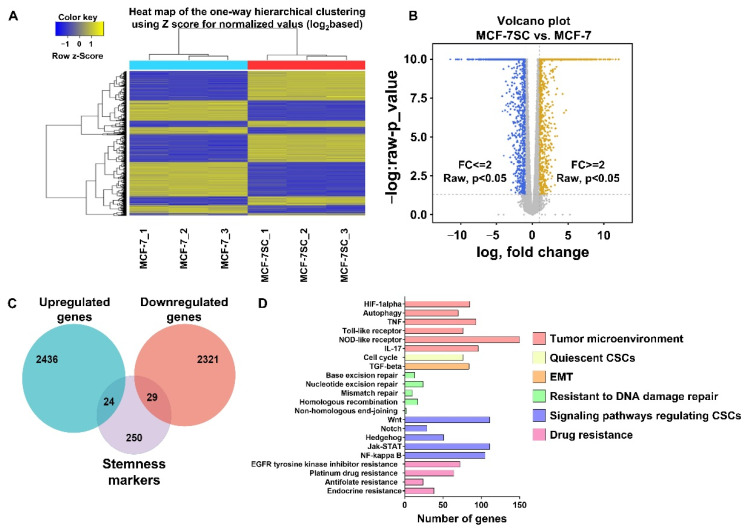
Transcriptome analysis of cultured MCF-7SCs. (**A**) Heat map of one-way hierarchical clustering using Z-score for normalized value (log2-based). (**B**) Volcano plot comparison of MCF-7 cells (fold change (FC) ≤ 2, *p* < 0.05) and MCF-7SCs (FC ≥ 2, *p* < 0.05). (**C**) Venn diagram showing the number of overlapped genes between upregulated and downregulated genes with stemness markers from a published database. (**D**) Representative stemness-related signaling pathways in MCF-7SCs compared with MCF-7 cells.

RNA-seq analysis was performed to evaluate the gene expression profiles of MCF-7SCs and MCF-7 cells (Figure 2A). As shown in Figure 2B, transcriptome analysis identified 2436 upregulated and 2321 downregulated genes (*p* < 0.05; fold difference > 2) in MCF-7SCs compared with MCF-7 cells. A Venn diagram was generated using The Cancer Genome Atlas (TCGA) database tool, which showed that 24 upregulated and 29 downregulated genes overlapped with 250 genes related to self-renewal pathways in CSCs from a published database [26] (Figure 2C). Moreover, a KEGG pathway analysis was conducted to examine signaling pathways implicated in the regulation of the stemness of MCF-7SCs. The identified pathways were associated with stemness regulation, including the tumor microenvironment, quiescent CSCs, the epithelial–mesenchymal transition (EMT), resistance to DNA damage repair, CSC signaling, and drug resistance (Figure 2D). CSCs resist DNA damage-induced cell death by nucleotide cleavage repair, mismatch repair, homologous recombination, and activation of a non-homologous end joining system [27]; these genes were activated in MCF-7SCs (Figure 2D). Signaling pathways involved in drug resistance—including EGFR tyrosine kinase inhibitor, platinum drugs, antifolate, and endocrine—were enriched in MCF-7SCs; these pathways are expected to promote cell stemness (Figure 2D). Therefore, the RNA-seq analysis of MCF-7SCs and MCF-7 cells indicated that the former have a distinct gene expression profile that was enriched in signaling pathways related to stemness characteristics.

### 3.3. MCF-7SCs Preferentially Used Glycolysis for Energy Generation

**Figure 3:**
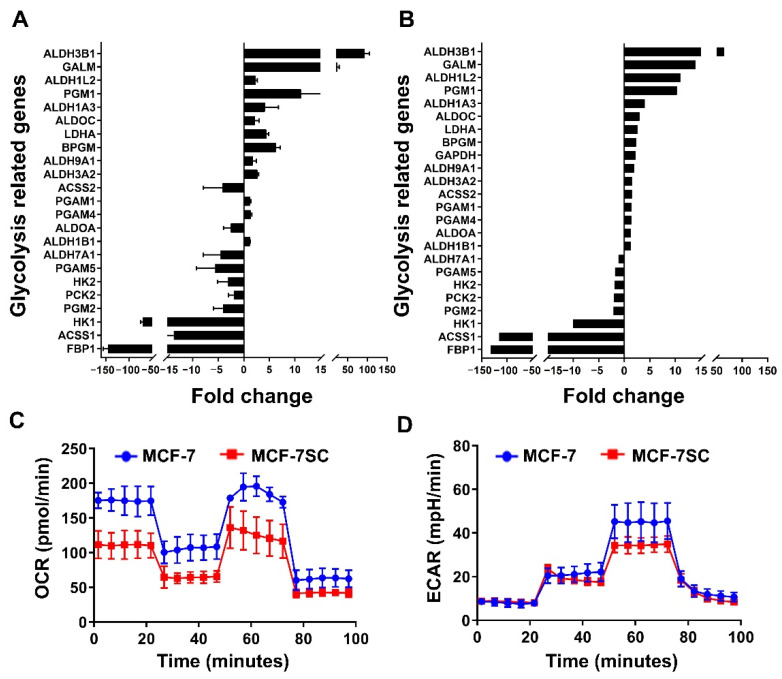

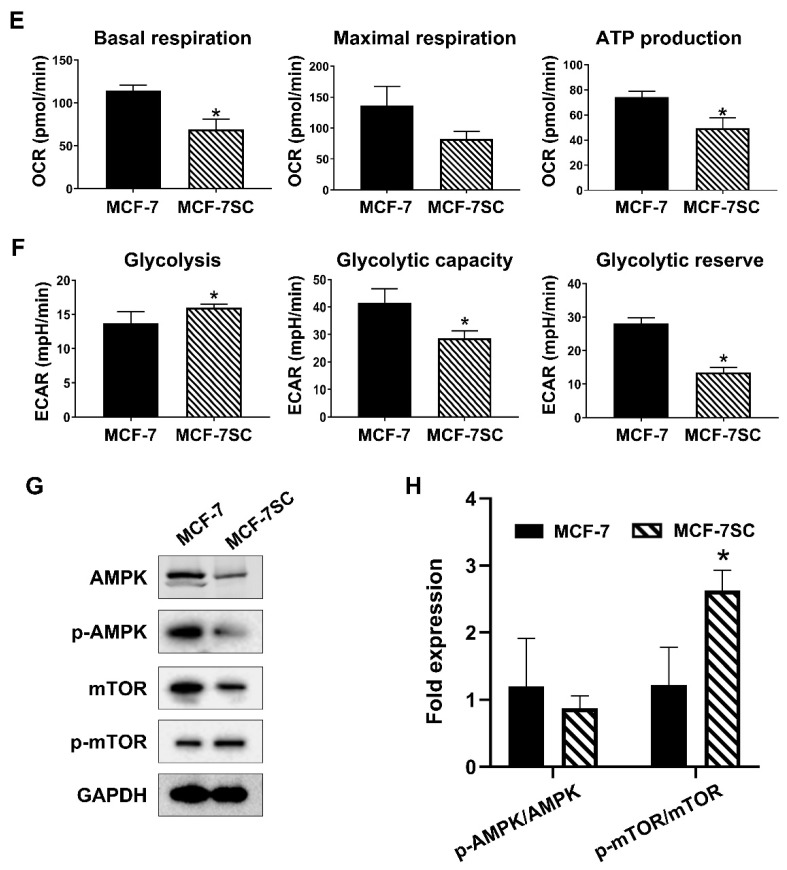
MCF-7SCs preferentially used glycolysis for energy production. (**A**) Up- and downregulated genes associated with glycolysis signaling. (**B**) Validation of the up- and downregulated genes shown in (**A**) using RT-qPCR. (**C**) Oxygen consumption rate (OCR) (pmol/min) and (**D**) extracellular acidification rate (ECAR) (mpH/min). (**E**) Basal respiration, maximal respiration, and ATP production levels. (**F**) Glycolysis, glycolytic capacity, and glycolytic reserve levels. (**G**) Expression of AMPK, p-AMPK, mTOR, and p-mTOR was detected by Western blotting. GAPDH was used as a loading control. (**H**) Quantification of p-AMPK/AMPK, and p-mTOR/mTOR expression normalized to GAPDH. * *p* < 0.05 vs. control; statistical values represent data from three biologically independent experiments.

Transcriptome analysis of MCF-7SCs and MCF-7 cells indicated an alteration in metabolism-related genes in MCF-7SCs, as listed in Table S2. Glucose is a major source of cellular energy. BCSCs have been reported to exhibit a distinct glucose metabolism, as they use glycolysis rather than OXPHOS to produce ATP [28]. Consistent with this observation, significant changes in genes related to glycolysis but not to OXPHOS were observed in MCF-7SCs (Table S2). As shown in Figure 3A, there were 16 upregulated and 8 downregulated genes related to glycolysis signaling. To validate the expression of these upregulated and downregulated glycolysis-related genes, RT-qPCR was performed. As shown in Figure 3B, with the exception of the acetyl-CoA synthetase 2 (ACSS2) and aldolase A (ALDOA) genes, expression of all of the glycolysis-related genes tested was consistent with the transcriptome analysis data. A Seahorse XF analyzer was used to measure the oxygen consumption rate (OCR) and the extracellular acidification rate (ECAR) in MCF-7 cells and MCF-7SCs (Figure 3C,D). While the OCR represents OXPHOS utilization, ECAR measures intracellular glycolysis activity [29]. Compared with MCF-7 cells, MCF-7SCs appeared to use glycolysis rather than OXPHOS to produce energy, as evidenced by higher glycolysis and lower basal respiration levels (Figure 3E,F). Reflecting the differences in energy metabolism between the two cell lines, transcriptome analysis revealed 95 and 129 genes related to the AMPK and mammalian target of rapamycin (mTOR) signaling pathways, respectively (Figure S2). Moreover, Western blotting revealed that the relative levels of p-AMPK/AMPK were similar between the two cells. Interestingly, the relative amounts of p-mTOR/mTOR were somewhat higher in MCF-7SC cells (Figure 3G,H). Therefore, MCF-7SCs use glycolysis rather than OXPHOS for energy generation (Figure 3), consistent with a prior report [28].

### 3.4. Nootkatone Inhibited the Proliferation of MCF-7SCs via AMPK Activation

**Figure 4:**
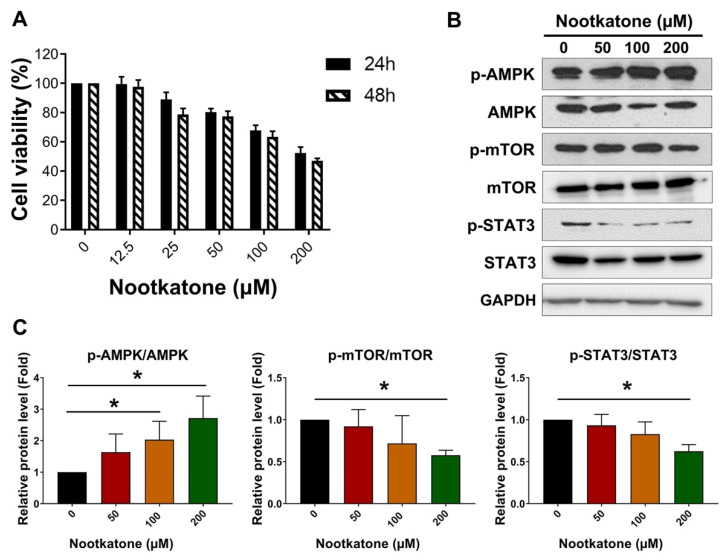

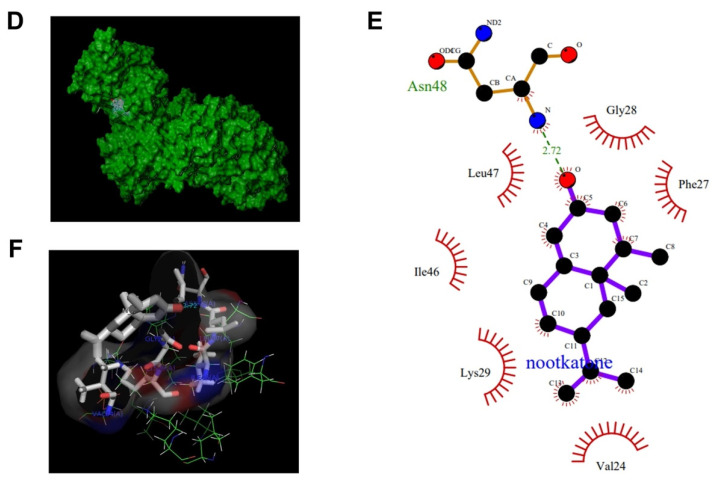
Nootkatone inhibited the proliferation of MCF-7SCs via AMPK activation. (**A**) The viability of MCF-7SCs was evaluated by MTT assay following nootkatone treatment for 24 h and 48 h. (**B**) Expression of AMPK, p-AMPK, mTOR, p-mTOR, STAT3, and p-STAT3 was detected by Western blotting following treatment with nootkatone for 48 h. GAPDH was used as a loading control. (**C**) Quantification of AMPK, p-AMPK, mTOR, p-mTOR, STAT3, and p-STAT3 expression normalized to GAPDH. (**D**) Image of the 30 nootkatone–AMPK complexes. (**E**) Interactions between nootkatone and AMPK analyzed using the LigPlot program. (**F**) Nootkatone–AMPK complex generated using the PyMol program. * *p* < 0.05 vs. control; statistical values represent data from three biologically independent experiments.

Nootkatone was previously identified as a potential AMPK activator in A549 lung cancer cells [19]. Thus, we hypothesized that nootkatone might also modulate AMPK signaling in BCSCs. First, we examined whether nootkatone could inhibit cell proliferation in BCSCs. According to MTT assay, nootkatone reduced the viability of MCF-7SCs after 24 and 48 h (Figure 4A). Notably, nootkatone induced p-AMPK/AMPK and reduced p-mTOR/mTOR and p-STAT3/STAT3, indicating the essential role of nootkatone in the activation of AMPK signaling (Figure 4B,C). To validate the role of nootkatone as an AMPK activator, in silico docking was performed. The 3D structure of AMPK was obtained from the Protein Data Bank (PDB). Because nootkatone has an octahydronaphthalene moiety, 4cfe.pdb was selected for in silico docking. The binding site of its ligand, 5-((6-chloro-5-(1-methyl-1H-indol-5-yl)-1H-benzo[d]imidazol-2-yl) oxy)-2-methylbenzoic acid (named 992) was determined using the LigPlot program as follows: (chain A) Val11, Leu18, Gly28, Lys29, Lys31, Ile46, Asn48, Asp88, Phe90, (chain B) Val81, Arg83, Thr106, Sep108, Asn111, Val113, Ile115 (Figure S3). The apo-protein of AMPK without 992 was prepared by the Sybyl program. After energy minimization, the root mean square deviation between the X-ray crystallographic structure and the apo-protein was 0.0069 Å. To validate the docking process, 992 was docked into the apo-protein. The radius for the flexible docking was set to 6.5 Å, the default in Sybyl. Because the current flexible docking procedure iterated 30 times, 30 ligand–protein complexes were generated. The binding energy ranged between −21.18 and −13.76 kcal/mol. The 3D structure of nootkatone was obtained from PubChem (https://pubchem.ncbi.nlm.nih.gov/, accessed on 20 September 2020), whose PubChem CID was 1268142. Like 992, 30 complexes between nootkatone and the apo-protein of AMPK were generated after 30 iterations. As shown in Figure 4D, 30 ligands were docked into the binding site [25]. The binding energy ranged between −7.20 and −5.41 kcal/mol. The complex showing the lowest binding energy was selected for analysis. The interactions between nootkatone and AMPK were analyzed using the LigPlot program. As shown in Figure 4E, Val24, Phe27, Gly28, Lys29, Ile46, and Leu47 participated in the hydrophobic interactions and Asn48 formed a hydrogen bond between ketone of nootkatone and hydrogen from amino group of peptide bond (2.72 Å). Sixteen residues participated in the interactions between 992 and AMPK, seven residues were involved in nootkatone. Comparing the structure of nootkatone with that of 992, nootkatone was found to be smaller than 992 (Figure S4). As shown in Figure 4F, nootkatone docked well with AMPK. Although the binding affinity of 992 was high at 2 nM EC_50_, that of nootkatone in vitro was at the micromolar level. This may result in the above binding energy. The in silico docking results demonstrated for the first time that nootkatone can be docked onto AMPK, suggesting an interaction. Taken together, these results suggest that nootkatone inhibits the proliferation of MCF-7SC BCSCs by directly binding to and activating AMPK.

### 3.5. Nootkatone Impaired Glucose Metabolism in MCF-7SCs

**Figure 5:**
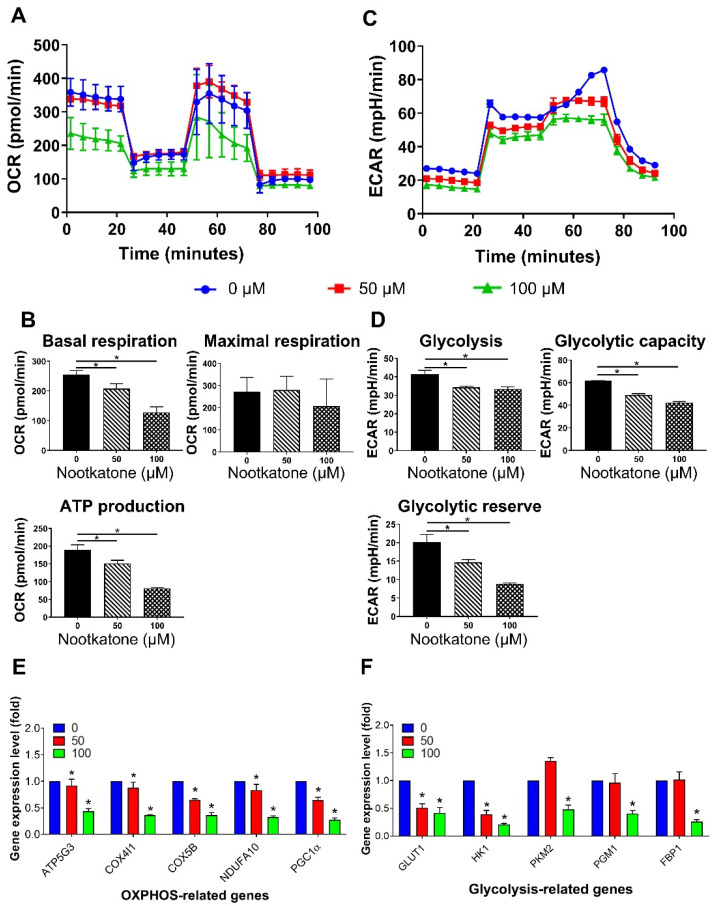
Nootkatone impaired glucose metabolism in MCF-7SCs. (**A**) Oxygen consumption rate (OCR) (pmol/min) was assessed following nootkatone treatment for 48 h. (**B**) Basal respiration, maximal respiration, and ATP production were obtained from OCR results. (**C**) The extracellular acidification rate (ECAR) (mpH/min) was measured following incubation for 48 h with nootkatone. (**D**) Glycolysis, glycolytic capacity, and glycolytic reverse obtained from ECAR results. (**E**) Expression of oxidative phosphorylation (OXPHOS)-related genes and (**F**) glycolysis-related genes was analyzed by RT-qPCR following nootkatone treatment for 48 h. * *p* < 0.05 vs. control; statistical values represent data from three biologically independent experiments.

Nootkatone disrupted glucose metabolism in MCF-7SCs, as evidenced by reductions of the OCR and ECAR (Figure 5A–D). Consistent with these results, decreased expression of OXPHOS-related genes [30,31,32], such as ATP synthase lipid-binding protein (ATP5G3), cytochrome c oxidase subunit 4I1 (COX4I1), cytochrome c oxidase subunit 5B (COX5B), NADH:ubiquinone oxidoreductase subunit A10 (NDUFA10), and peroxisome proliferator-activated receptor gamma coactivator 1-alpha (PGC-1α), was observed after nootkatone treatment (Figure 5E). Moreover, reduction of ECAR by nootkatone was supported by decreased expression of genes related to glycolysis, such as glucose transporter 1 (GLUT1), hexokinase (HK1), pyruvate kinase 2 (PKM2), phosphoglucomutase (PGM1), and fructose-bisphosphatase 1 (FBP1) (Figure 5F). Overall, our results demonstrate that nootkatone impairs glucose metabolism in MCF-7SCs.

### 3.6. Nootkatone Reduced the Stemness of MCF-7SCs

**Figure 6:**
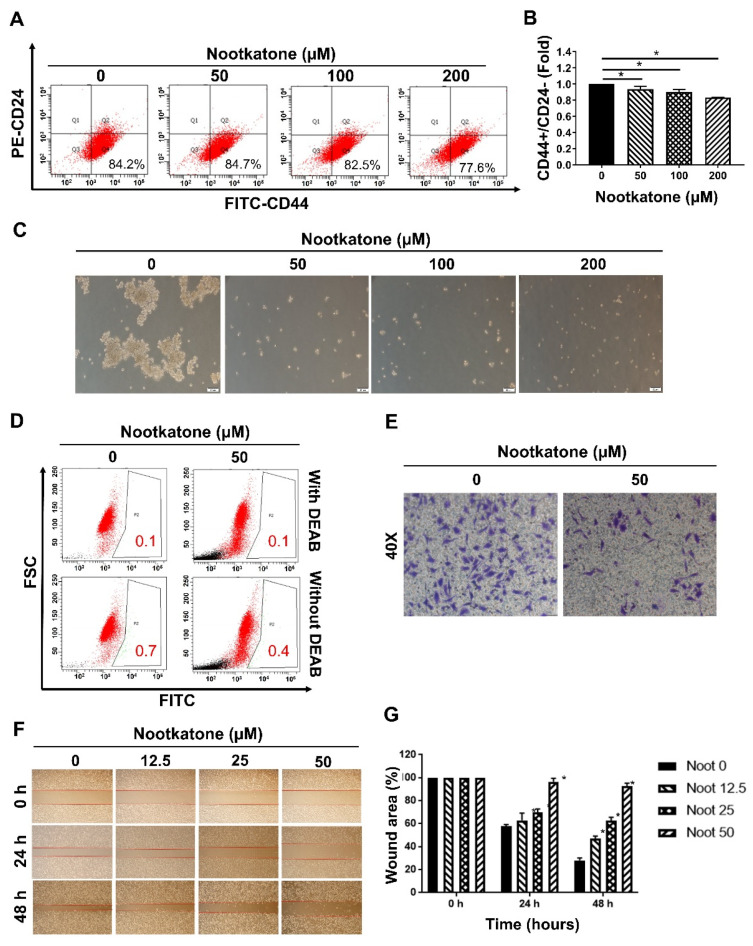
Nootkatone reduced the stemness of MCF-7SCs. (**A**,**B**) CD44^+^/CD24^−^ populations were measured by FACS following nootkatone treatment for 48 h. (**C**) Mammospheres were cultured in complete MammoCult Human Medium (magnification, 100×) for 10 days. (**D**) The aldehyde dehydrogenase (ALDH^+^) population was determined using an ALDEFLUOR assay kit following nootkatone treatment for 48 h. DEAB was used as a negative control. (**E**) Invasion was evaluated using an invasion assay. Cells were treated with or without 50 μM nootkatone for 48 h (100× magnification). (**F**,**G**) Migration capacity was evaluated using a wound-healing assay following nootkatone treatment for 24 h and 48 h. * *p* < 0.05 vs. control; statistical values represent data from three biologically independent experiments.

FACS analysis demonstrated that nootkatone dramatically reduced the CD44^+^/CD24^−^ cell population of MCF-7SCs (Figure 6A,B). Mammosphere formation of MCF-7SCs was significantly diminished by nootkatone treatment in a dose-dependent manner (Figure 6C). In addition, ALDH activity was dramatically reduced by non-lethal concentrations of nootkatone (Figure 6D). We next examined whether nootkatone could affect the migration and invasive abilities of MCF-7SCs using wound healing and chamber assays. At non-lethal concentrations, the migration and invasion abilities of MCF-7SCs were suppressed by nootkatone (Figure 6E–G). Taken together, these results show that nootkatone inhibits the stemness of MCF-7SCs in a dose-dependent manner.

## 4. Discussion

CSCs play critical roles in the development of chemo- or radioresistance and cancer recurrence [33,34]. CSCs possess characteristics such as the ability to self-renew and differentiate into heterogeneous phenotypes, as well as the capacity to initiate and maintain tumor development [34,35]. In a previous study in our laboratory, MCF-7SC breast cancer cells were found to possess an increased population of CD44^+^/CD24^−^ cells, higher ALDH levels, reduced ROS levels, overexpressed drug efflux proteins (MDR1 and MRP1), increased mammosphere formation capacity, and increased migration and invasion [3]. To further confirm the stem cell properties of MCF-7SCs in vivo, we focused on tumorigenic capacity in a xenograft model. Indeed, the stemness of MCF-7SCs was confirmed by their strong ability to form tumors in a xenograft mouse model (Figure 1). Our findings demonstrate that MCF-7SCs exhibit characteristics of BCSCs.

Recently developed biological techniques have made it possible to simultaneously identify large numbers of genes at the genome level [36]. One of the most widely used high-throughput techniques for examining cellular RNAs is transcriptome analysis [37]. To evaluate the characteristics of MCF-7SCs at the genetic level, we performed a transcriptomic analysis, which revealed overexpression of signaling pathways involved in self-renewal and differentiation processes, such as Wnt/β-catenin, JAK/STAT3, NF-κB, Notch, and Hedgehog in MCF-7SCs. Enrichment of HIF-1α and autophagy-related signaling pathways was also observed, indicating an alteration in the MCF-7SC tumor environment, as HIF-1α is involved in the regulation and maintenance of CSCs in hypoxic microenvironments [38]. Alterations in the tumor environment were further supported by the enrichment of inflammatory-related signaling pathways including tumor necrosis factor (TNF), Toll-like receptor, and interleukin (IL)-17, which are overexpressed in CSCs [39]. As EMT activation via transforming growth factor (TGF)-β can confer stem properties to cancer cells [40], TGF was also activated in our MCF-7SCs. In particular, CSCs retain resistance to DNA-damage-induced cell death through nucleotide cleavage repair, nucleotide cleavage repair, mismatch repair, homologous recombination, and activation of a non-homologous end joining system [27]; activation of these genes was also observed in MCF-7SCs. Signaling pathways involved in drug resistance, including the EGFR tyrosine kinase inhibitor, platinum drugs, antifolate, and endocrine, were enriched in MCF-7SCs; these pathways are expected to promote cell stemness.

Transcriptome analysis of MCF-7SCs revealed a significant change in the expression of genes associated with glycolysis, but not in that of genes associated with OXPHOS (Table S2). Interestingly, among the eight downregulated genes, the expression of fructose-1,6-bisphosphatase 1 (FBP1) was 150-fold reduced compared to MCF-7 cells. These results are consistent with a report of a negative correlation between FBP1 expression and the survival rate of patients with breast cancer. Furthermore, a previous study reported the critical role of FBP1 in the metabolism of breast cancer cells. Downregulation of FBP1 enhanced glucose uptake by promoting glycolysis [8]. The ALDH superfamily plays a critical role in cancer metabolism by upregulating the glucose transporter 1 (GLUT1), enhancing glycolysis [41]. Aldehyde dehydrogenase 3 family member B1 (ALDH3B1) is overexpressed in many human cancer types [42]; increased expression of ALDH3B1 was also observed in MCF-7SCs. We observed a significant enrichment of the AMPK and mTOR signaling pathways, which regulate glucose metabolism. BCSCs exhibit distinct glucose metabolic machinery, and glycolysis is considered the major source of energy production. A Seahorse XF analyzer was used to measure the OCR and ECAR in MCF-7 cells and MCF-7SCs; the results confirmed that MCF-7SCs used glycolysis rather than OXPHOS for energy, consistent with a previous study [28].

Nootkatone is a bioactive ingredient in grapefruit that exerts anticancer effects in lung and colorectal cancer. Incubation with nootkatone has been reported to induce AMPK activation, resulting in suppression of the Erk and Akt signaling pathways in A549 cells. Moreover, nootkatone treatment inhibited cell proliferation in colorectal cancer cells, as evidenced by a reduction in cyclin D1 [19,20]. On the other hand, nootkatone has also been well characterized as an AMPK activator in vitro and in vivo. Nootkatone activates AMPK; this effect is mediated by LKB1 and CaMKK in the mouse muscle myoblast cells C2C12. Moreover, enhanced AMPK activity along with an elevated AMP/ATP ratio induced phosphorylation of the downstream target acetyl-CoA carboxylase (ACC). Notably, administration of nootkatone at a concentration of 200 mg/kg in a mouse model dramatically induced AMPK activation together with LKB1 and ACC phosphorylation in the liver and muscle of mice [18]. AMPK can be indirectly activated by numerous modulators that cause AMP or calcium accumulation. These modulators, including metformin, troglitazone, rosiglitazone, resveratrol, quercetin, berberine, and curcumin, activate LKB1 and CaMKK, mediators of AMP or calcium accumulation [43]. By contrast, several other AMPK activators can directly bind and alter the conformation of a specific AMPK subunit, leading to activation of AMPK without any alteration in ATP, ADP, or AMP levels. The first direct AMPK activator is 5-aminoimidazole-4-carboxamide riboside (AICAR), which can generate AICAR monophosphate (ZMP) [44]. With a structure similar to AMP, ZMP can interact with the γ-subunit of AMPK. Similar to AICAR, multiple compounds, including salicylate (known as the pro-drug of aspirin), benzimidazole (compound 911), compound-13, and MT63-78, have been reported to exert high specificity for the β-subunit of AMPK [45,46,47]. An in vitro study revealed that PT-1 has high affinity for AMPK complexes containing the γ-subunit [48]. We report for the first time that nootkatone interacts with AMPK. Notably, nootkatone treatment (50 and 100 μM) impaired glucose metabolism in MCF-7SCs, as evidenced by reduced glycolysis, possibly due to activation of AMPK. Taken together, our results suggest the possibility of direct activation of AMPK by interaction with nootkatone.

Targeting stem cell populations in tumors is considered an important strategy to increase the effectiveness of cancer treatments [49]. To date, no study has evaluated the effect of nootkatone in BCSCs. The CD44^+^/CD24^−^ population was possessed as a specific surface marker for breast cancer stem cell identification, promoting cell proliferation and metastasis [50]. A previous study identified a subpopulation with CD44^+^/CD24^−^ phenotype that was sorted from breast patient samples and exerted a strong tumorigenic capacity in vivo. Specifically, a very low number of CD44^+^/CD24^−^ cells (100 cells) can form the tumor, whereas up to 10,000 non CD44^+^/CD24^−^ cells failed to form tumors in NOD/SCID mice. Therefore, targeting the CD44^+^/CD24^−^ population could be considered one of the promising therapeutic therapies for breast cancer patients [51]. In this paper, nootkatone (50, 100, and 200 μM) could reduce CD44^+^/CD24^−^, indicating the vital role of nootkatone in the suppression of this tumorigenic cell population. The ability of nootkatone to inhibit the stem cell properties of MCF-7SCs was further confirmed by decreases in the ALDH^+^ populations (concentration used 50 μM) and by decreased mammosphere formation (concentration used 50, 100, and 200 μM) following nootkatone treatment. Convincing evidence has shown the role of BCSCs in metastasis and the close relationship between BCSCs and metastasis [52]. Invasion and wound healing assays showed that nootkatone suppressed the metastasis of MCF-7SCs. Together, our results provide a novel approach to target CSC populations using nootkatone to increase the effectiveness of breast cancer treatments. Moreover, there was a positive correlation between type 2 diabetes mellitus and breast cancer, as there was 15% of diabetes mellitus women patients could subsequently develop advanced stage of breast cancer [53]. Interestingly, Guo et al. revealed that nootkatone could inhibit the development of type 2 diabetes mellitus, as evidenced by the suppression of the α-glucosidase activity after nootkatone treatment in vitro [54]. However, the effect of nootkatone treatment on a diabetes mellitus mouse model remained unclear up to date. Therefore, further studies are needed to elucidate the efficacy of nootkatone on a mouse model harboring both diabetes mellitus and breast cancer.

## 5. Conclusions

In conclusion, MCF-7SCs, BCSCs isolated from parental MCF-7 cells, use glycolysis rather than OXPHOS for their energy supply. Furthermore, for the first time, our data show that nootkatone, a bioactive component of grapefruit, impairs glucose metabolism through AMPK activation and reduces the stemness of MCF-7SCs. The interaction between nootkatone and the active site of AMPK was shown using in silico docking analysis. This study proposes a novel approach using nootkatone to regulate glucose metabolism and target CSC populations to increase the effectiveness of breast cancer treatments.

## Data Availability

All data are available within the article and in the Supplementary Materials.

## Supplementary Materials

The following supporting information can be downloaded at: https://www.mdpi.com/article/10.3390/pharmaceutics14050906/s1, Figure S1: Metastasized tumors from BALB/C nude mice injected with MCF-7SCs. Figure S2: The AMPK/mTOR signaling pathway was enriched in MCF-7SCs. Figure S3: Interactions between 992 contained in 4cfe.pdb and AMPK analyzed using the LigPlot program. Figure S4: The structures of nootkatone (top) and 5-((6-chloro-5-(1-methyl-1H-indol-5-yl)-1H-benzo[d]imidazol-2-yl)oxy)-2-methylbenzoic acid (named as 992) (bottom). Table S1: Sequences of RT-qPCR primers. Table S2: Metabolism-related genes (Microsoft Excel™ file).

## Abbreviations

ACC	Acetyl-CoA carboxylase
AICAR	5-Aminoimidazole-4-carboxamide riboside
ALDH	Aldehyde dehydrogenases
AMPK	AMP-activated protein kinase
BCSCs	Breast cancer stem cells
CaMKK	Calcium/calmodulin-dependent protein kinase
CSCs	Cancer stem cells
DEAB	Dimethylaminobenzaldehyde
ECAR	Extracellular acidification rate
EMT	Epithelial–mesenchymal transition
FACS	Fluorescence-activated cell sorting
FBS	Fetal bovine serum
FC	Fold change
IgG	Immunoglobulin G
IL	Interleukin
KEGG	Kyoto Encyclopedia of Genes and Genomes
LKB1	Liver kinase B1
MRP1	Multidrug resistance-associated protein 1
mTOR	Mammalian target of rapamycin
MTT	3-(4,5-Dimethylthiazol-2-yl)-2,5-diphenyl-tetrazolium bromide
OCR	Oxygen consumption rate
OXPHOS	Oxidative phosphorylation
PCR	Polymerase chain reaction
PDB	Protein data bank
RIPA	Radioimmunoprecipitation assay
RT-qPCR	Quantitative reverse transcription PCR
TCGA	The Cancer Genome Atlas
TGF	Transforming growth factor
TNF	Tumor necrosis factor
ZMP	5-Aminoimidazole-4-carboxamide riboside monophosphate
