# Supplementary material for: Impairment of Glucose Metabolism and Suppression of Stemness in MCF-7/SC Human Breast Cancer Stem Cells by Nootkatone
Source: Pharmaceutics. 2022 Apr 21;14(5):906. doi: 10.3390/pharmaceutics14050906 (PMC9145028; doi:10.3390/pharmaceutics14050906)
Supplement: Supplementary file 1 [file pharmaceutics-14-00906-s001.zip › pharmaceutics-1659800-supplementary.pdf]

pharmaceutics

Article

Supplementary Materials: Impairment of Glucose Metabolism and Suppression of Stemness in MCF-7/SC Human Breast Cancer Stem Cells by Nootkatone

Yen Thi-Kim Nguyen, Ngoc Bao To, Vi Nguyen-Phuong Truong, Hee Young Kim, Meran Keshawa Ediriweera, Yoongho Lim and Somi Kim Cho

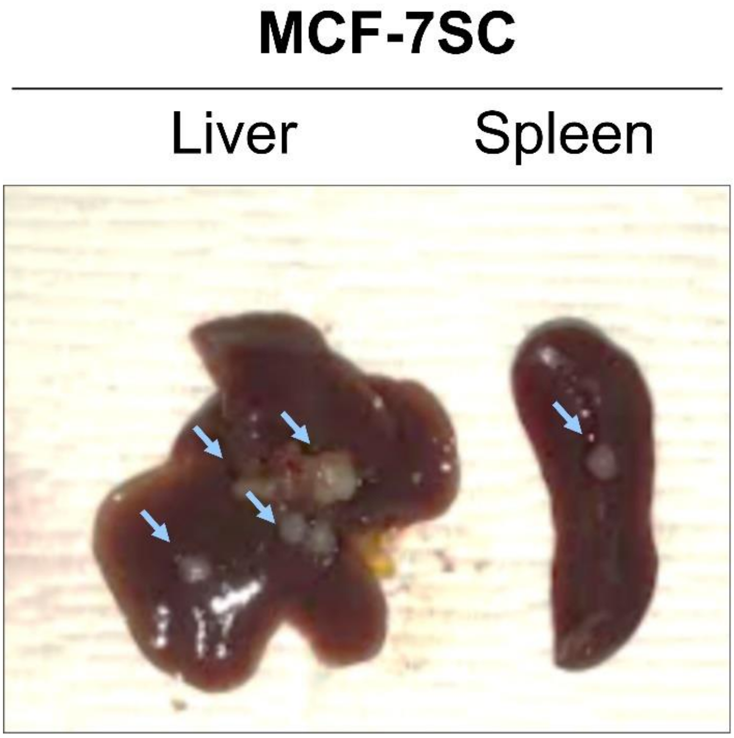

Figure S1. Metastasized tumors from BALB/C nude mice injected with MCF-7SCs (light blue arrows; to liver and spleen).

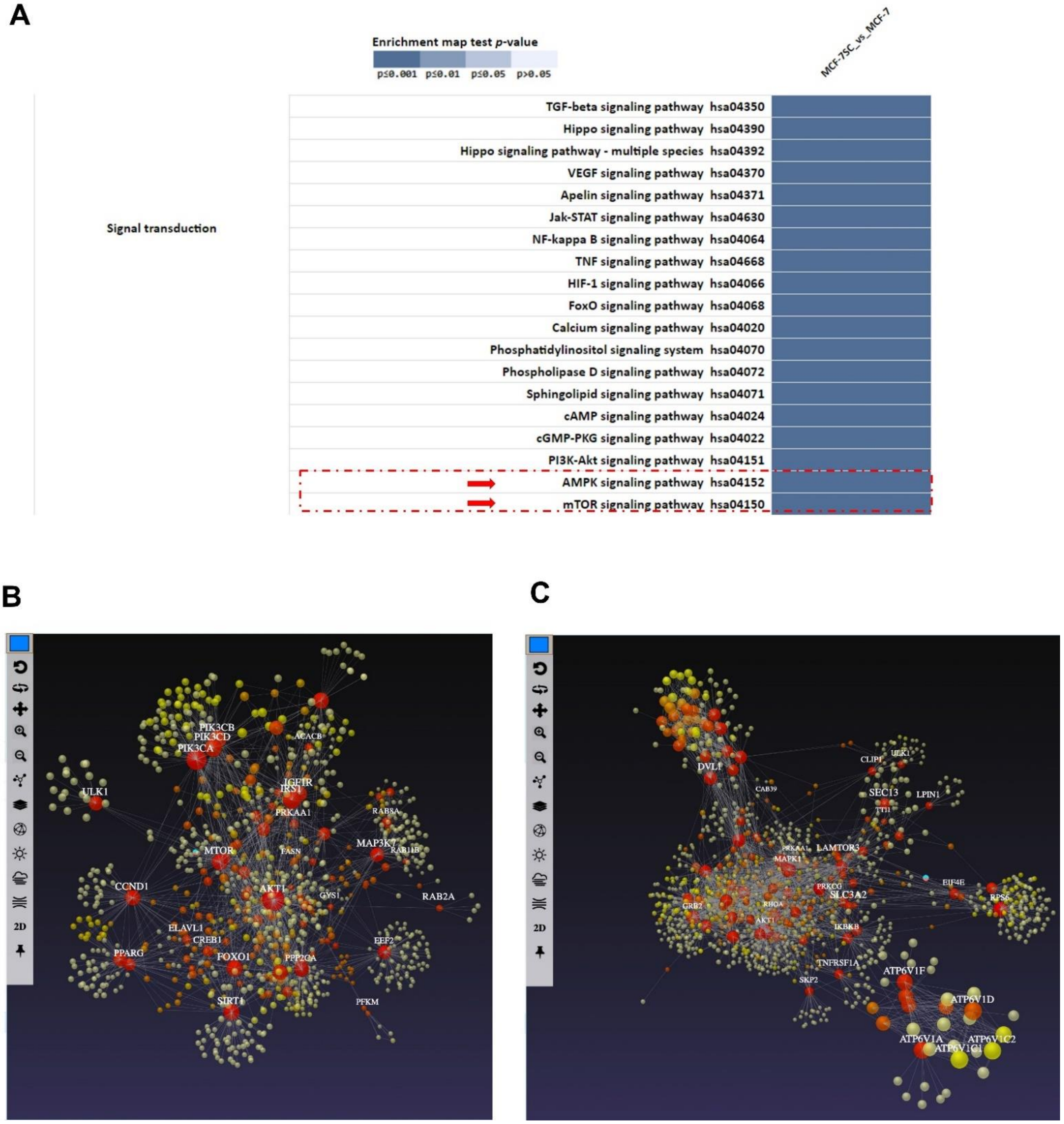

Figure S2. The AMPK/mTOR signaling pathway is enriched in MCF-7SCs. (**A**) KEGG pathways determined by transcriptomic analysis. (**B**) Enrichment of the AMPK and (**C**) mTOR signaling pathways analyzed using the NetworkAnalyst tool.

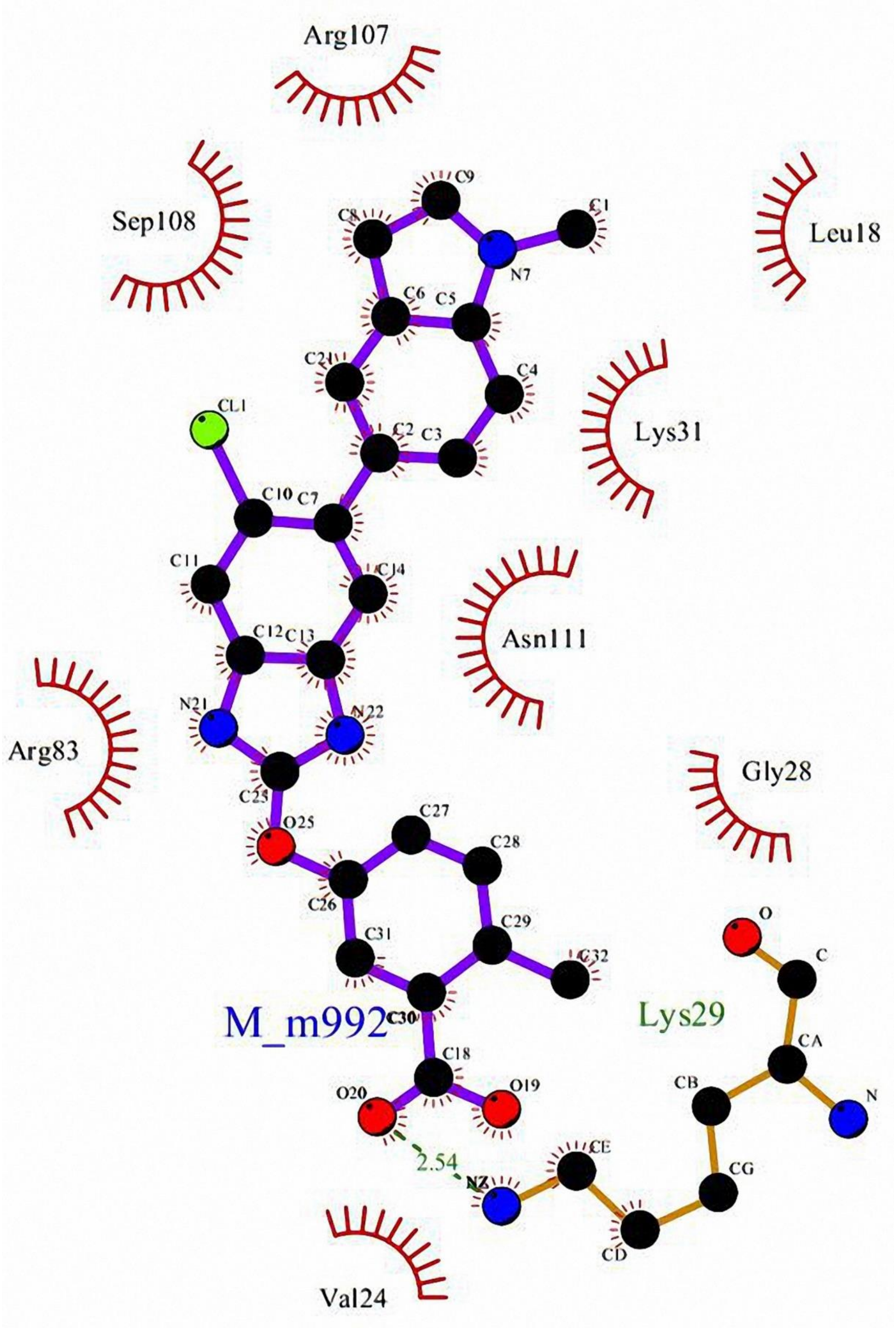

Figure S3. Interactions between 992 contained in 4cfe.pdb and AMPK analyzed using the LigPlot program.

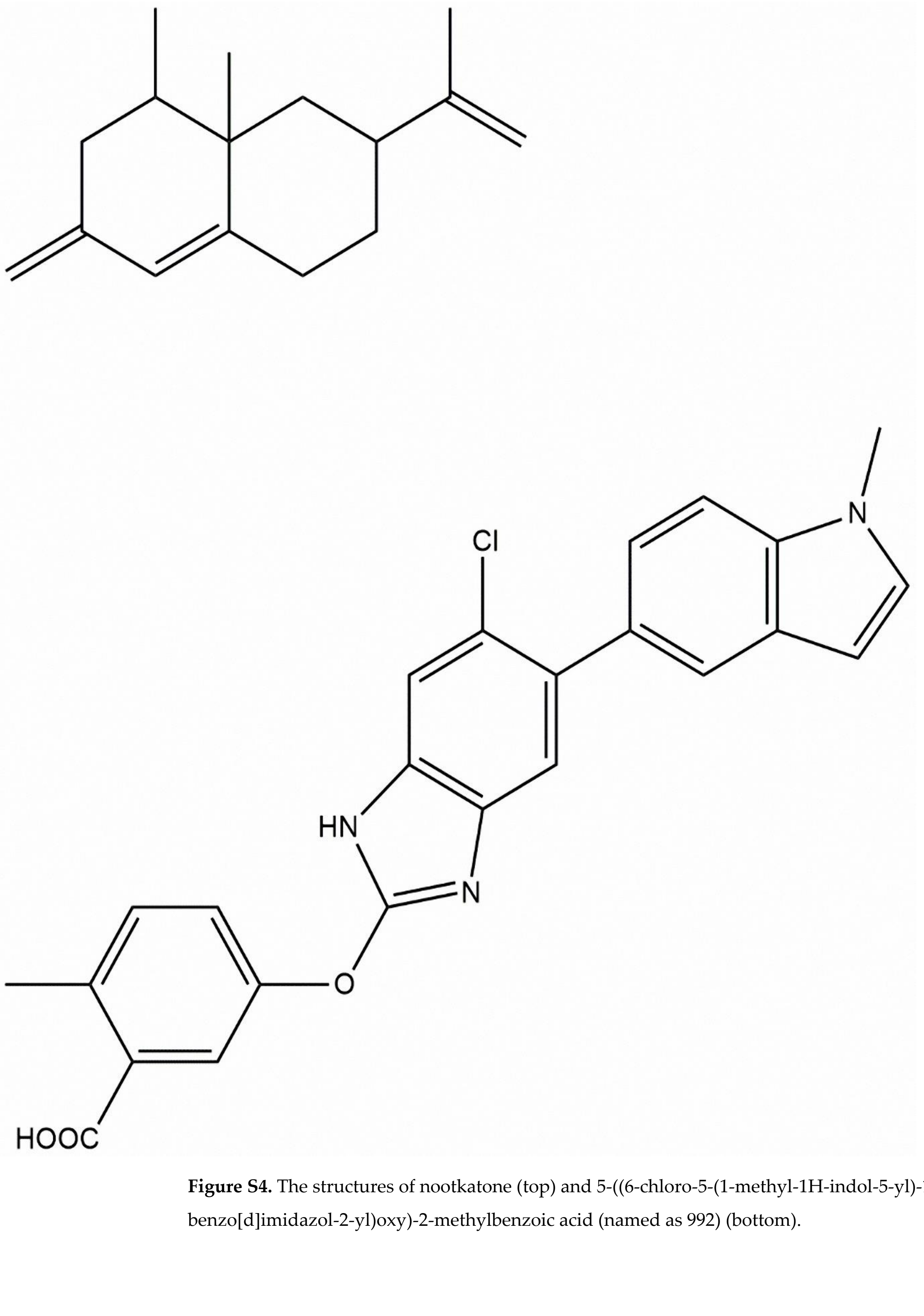

Figure S4. The structures of nootkatone (top) and 5-((6-chloro-5-(1-methyl-1H-indol-5-yl)-1H-benzo[d]imidazol-2-yl)oxy)-2-methylbenzoic acid (named as 992) (bottom).

Table S1. Sequences of RT-qPCR primers

Genes		Primer sequences
**ALDH3B1**	**Forward (5′->3′)**	TGCTATGTGGATGACAACTGCG
	**Reverse (5′->3')**	CGTGTGATGGCATTCTGTAGGG
**GALM**	**Forward (5′->3′)**	CAACCGAATCGCCAAAGGAACC
	**Reverse (5′->3')**	CGAGAACTGGACGCCATTTGAC
**ALDH1L2**	**Forward (5′->3′)**	GCCTGGTCTCGTTACCAAA
	**Reverse (5′->3')**	GCCACTTTCACCTCTTCAGC
**PGM1**	**Forward (5′->3′)**	TGATGGACGCGAGCAAACTGTC
	**Reverse (5′->3')**	ATGTCCTCCACACTCTGCTTGC
**ALDH1A3**	**Forward (5′->3′)**	GCCCTTTATCTCGGCTCTCT
	**Reverse (5′->3′)**	CGGTGAAGGCGATCTTGT
**ALDOC**	**Forward (5′->3′)**	AAATTGGGGTGGAAAACACA
	**Reverse (5′->3′)**	AGAAAATGACGCCTCCAATG
**LDHA**	**Forward (5′->3′)**	TGGGAGTTCACCCATTAAGC
	**Reverse (5′->3′)**	AGCACTCTCAACCACCTGCT
**BPGM**	**Forward (5′->3′)**	GGAAATAGCAGTAGGGCACTCC
	**Reverse (5′->3′)**	CAACAGCACGCAGGTTTTCATCC
**ALDH9A1**	**Forward (5′->3′)**	AGCTGAAGACGGTGTGTGTG
	**Reverse (5′->3')**	CCCAAAGCCTGGATGTAAGA
**ALDH3A2**	**Forward (5′->3′)**	ACTGATAGGAGCCATCGCTGCA
	**Reverse (5′->3′)**	GCTCCGTGGTTTCCTCAACACC
**ACSS2**	**Forward (5′->3′)**	CGAGGCCCTGCAGAAGTGTC
	**Reverse (5′->3′)**	GAGTCACCCATGCCGAGCTC
**PGAM1**	**Forward (5′->3′)**	GGAGGGGAAACGTGTACTGA
	**Reverse (5′->3′)**	CAGAGAGACCCTCCAGATGC
**PGAM4**	**Forward (5′->3′)**	CGCTACGAGATGCTGGCTATGA
	**Reverse (5′->3')**	CCATAGTGCCGCTCATTGAGGC
**ALDOA**	**Forward (5′->3′)**	GACACTCTACCAGAAGGCGGAT
	**Reverse (5′->3′)**	GGTGGTAGTCTCGCCATTTGTC
**ALDH1B1**	**Forward (5′->3′)**	CTGGAGCTGGGTGGTAAGAG
	**Reverse (5′->3′)**	CTTTCTCCACGGTTCTCTCG
**ALDH7A1**	**Forward (5′->3′)**	CAACGAGCCAATAGCAAGAG
	**Reverse (5′->3′)**	GCATCGCCAATCTGTCTTAC
**PGAM5**	**Forward (5′->3′)**	GAACTACATCCACCGAGCTGA
	**Reverse (5′->3′)**	GGGAAACTGCAACGCTCTAC
**HK2**	**Forward (5′->3′)**	AAGGCTTCAAGGCATCTG
	**Reverse (5′->3′)**	CCACAGGTCATCATAGTTCC
**PCK2**	**Forward (5′->3′)**	TAGTGCCTGTGGCAAGACCAAC
	**Reverse (5′->3′)**	GAAGCCGTTCTCAGGGTTGATG
**PGM2**	**Forward (5′->3′)**	CGGATGCTGATAGACTTGCTGTG
	**Reverse (5′->3')**	TGAGAGCACTGCGATCCTGGTT

**HK1**	**Forward (5′->3′)**	GGTGAAATCGTCCGCAAC
	**Reverse (5′->3')**	CCCGGGTCTTCATCGTC
**ACSS1**	**Forward (5′->3′)**	ATGGGCAGTGAGGACATGCTCT
	**Reverse (5′->3')**	CACAGCCAAAGATGTCACCTGG
**FBP1**	**Forward (5′->3′)**	TCAACTGCTTCATGCTGGAC
	**Reverse (5′->3')**	GGGTCAAAGTCCTTGGCATA
**GLUT1**	**Forward (5′->3′)**	GCTTCCTGCTCATCAATCGTAA
	**Reverse (5′->3')**	CGACCCTCTTCTTTCATCTCCT
**PKM2**	**Forward (5′->3′)**	ATCGTCCTCACCAAGTCTGG
	**Reverse (5′->3')**	GAAGATGCCACGGTACAGGT
**PGM1**	**Forward (5′->3′)**	AGCATTCCGTATTTCCAGCAG
	**Reverse (5′->3')**	GCCAGTTGGGGTCTCATACAAA
**ATP5G3**	**Forward (5′->3′)**	GGATTTGCCTTGTCTGAAGC
	**Reverse (5′->3')**	CGTACATTCCCATGACACCA
**COXAI1**	**Forward (5′->3′)**	ACGAGCTCATGAAAGTGTTGTG
	**Reverse (5′->3')**	AATGCGATACAACTCGACTTTCTC
**COX5B**	**Forward (5′->3′)**	CAGAAGGGACTGGACCCATA
	**Reverse (5′->3')**	TTCACAGATGCAGCCCACTA
**NDUFA10**	**Forward (5′->3′)**	CACCTGCGATTACTGGTTCAG
	**Reverse (5′->3')**	GCAGCTCTCTGAACTGATGTA
**PGC1α**	**Forward (5′->3′)**	TGCTAAACGACTCCGAGAA
	**Reverse (5′->3')**	TGCAAAGTTCCCTCTCTGCT
